# Author Correction: North Atlantic variability and its links to European climate over the last 3000 years

**DOI:** 10.1038/s41467-018-03338-1

**Published:** 2018-02-15

**Authors:** Paola Moffa-Sánchez, Ian R. Hall

**Affiliations:** School of Earth and Ocean Sciences, Cardiff University, CF10 3YE Cardiff, UK

Correction to: *Nature Communications* 10.1038/s41467-017-01884-8, published online 23 November 2017

In the original version of this Article, the third sentence of the first paragraph of the “Changes in the input of polar waters into the Labrador Sea” section of the Results originally incorrectly read “During the spring-summer months, after the winter convection has ceased in the Labrador Sea, its northwest boundary currents (the EGC and IC) support restratification of the surface ocean through lateral transport.” The correct version states “northeast” instead of “northwest”.

The fifth sentence of the second paragraph of the same section originally incorrectly read “In contrast, in the western section of the Nordic Seas, under the presence of warm Atlantic waters of the Norwegian Current, Nps was found to calcify deeper in the water column (100–200 m), whereas in the east under the influence of the EGC polar waters it calcified closer to the surface at a similar depth as Tq^23^.” The correct version states “eastern” instead of “western” and “west” instead of “east”.

The seventh sentence of the same paragraph originally incorrectly read “Small/large differences in Δδ^18^O_Nps-Tq_ indicating increased/decreased presence of warm and salty Atlantic IC waters vs. polar EGC waters in the upper water column, respectively.” The correct version starts ‘Large/small’ rather than “Small/large”.

These errors have been corrected in both the PDF and the HTML versions of the Article.

