# Supplementary material for: North Atlantic variability and its links to European climate over the last 3000 years
Source: Nat Commun. 2017 Nov 23;8:1726. doi: 10.1038/s41467-017-01884-8 (PMC5700112; doi:10.1038/s41467-017-01884-8)
Supplement: Supplementary file 1 — Supplementary Information [file 41467_2017_1884_MOESM1_ESM.pdf]

Supplementary Information

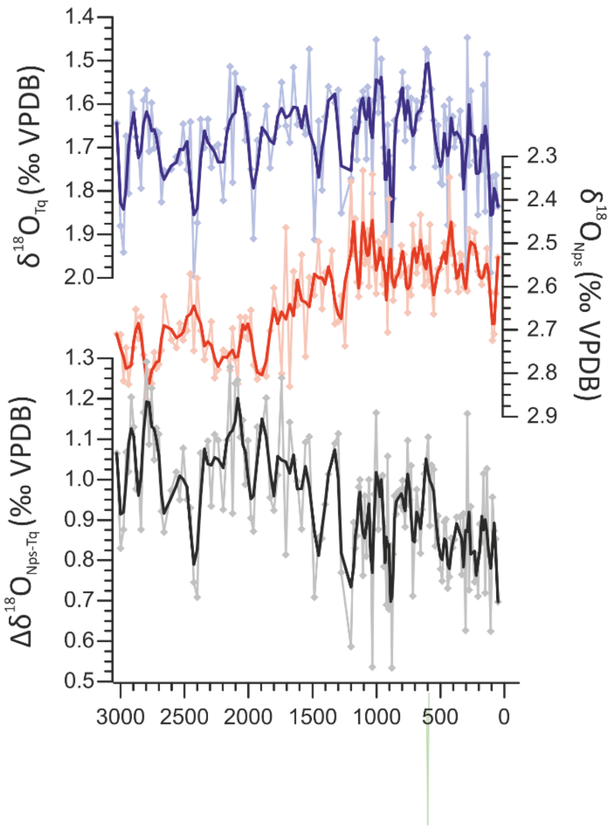

Supplementary Figure 1. Oxygen isotope data from core RAPiD-35-COM from *T. quinqueloba* (blue) and *N. pachyderma* (red). The difference of these two values ($\Delta\delta^{18}O_{Nps\text{-}Tq}$) is presented in black and in the Fig.2 and 3 (Note that there is a linear trend in the composition of the $\delta^{18}O$ Nps towards warmer/fresher conditions from 3000 years BP to the present. It is possible that this millennial scale trend is a result of the slight shift in blooming seasons [1] modified by the precession driven decrease in the summer insolation in the high northern latitudes).

Supplementary Note 1. Temperature and Salinity relationship in the Labrador Sea
The two proxies presented from site RAPiD-35-COM have a strong thermal component, however we argue for salinity to play a bigger role in the preconditioning for convection. Supplementary Figures 2 and 3 present temperature and salinity hydrographic data between 1940-2013 of the top 150 (taken mostly in spring/summer) (*courtesy of I. Yashayaev*) and illustrate that over the last 60 years the temperature and salinity in the surface Labrador Sea present a positive relationship (R=0.63 annually, R=0.86 decadally, Supplementary Figure 3). This relationship strongly suggests that at multidecadal time-scales our reconstructed changes in temperature will be accompanied with a similar trend in the salinity. For a broader view of these anomalies in the top 200m please refer to Figure 3a and 3b in [2].

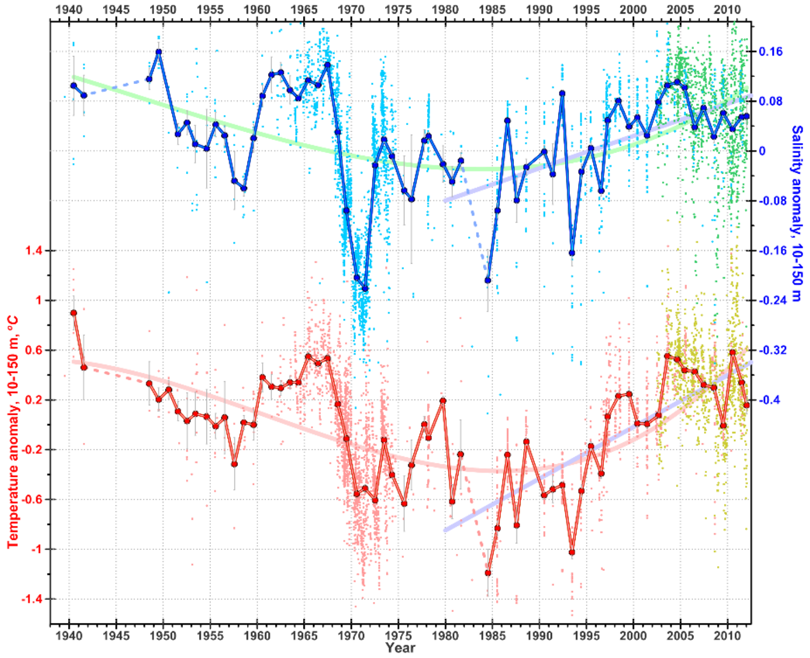

Supplementary Figure 2. Temperature and Salinity time-series from the top 150m in the Labrador Sea comprising AR7 and Argo *(Courtesy of I. Yashayaev)*. The data is presented as anomalies from the mean.

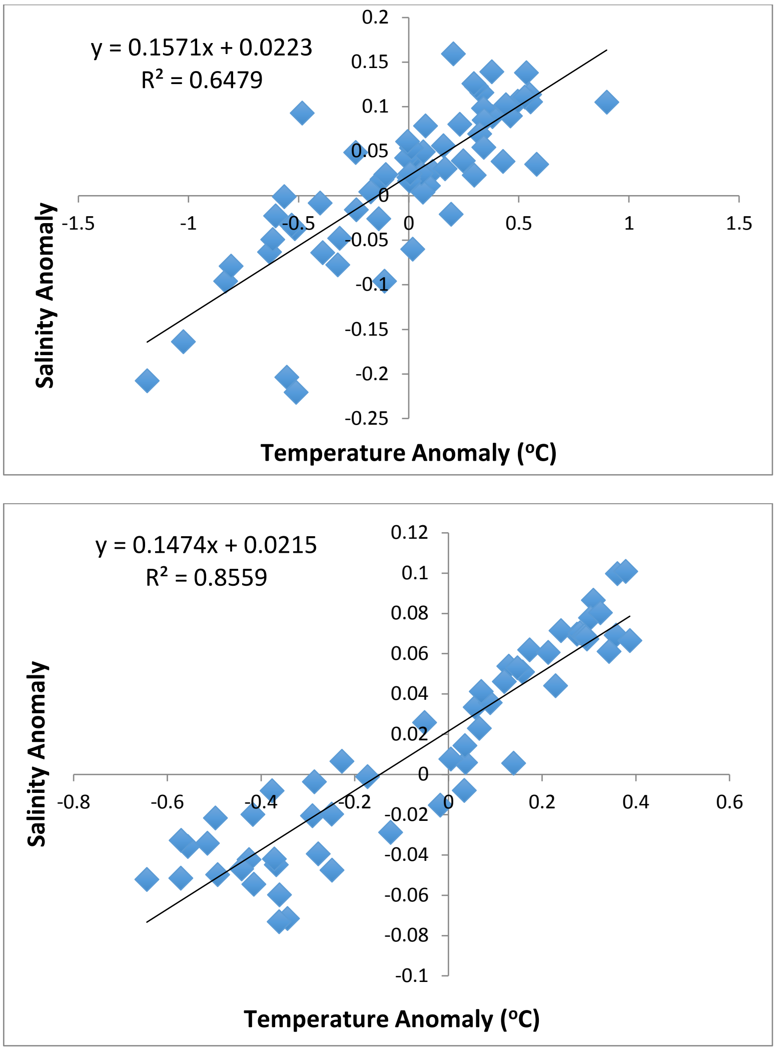

Supplementary Figure 3. Annual (top) and 9-point running mean (bottom) temperature and salinity data from the time-series presented Supplementary Figure 2.

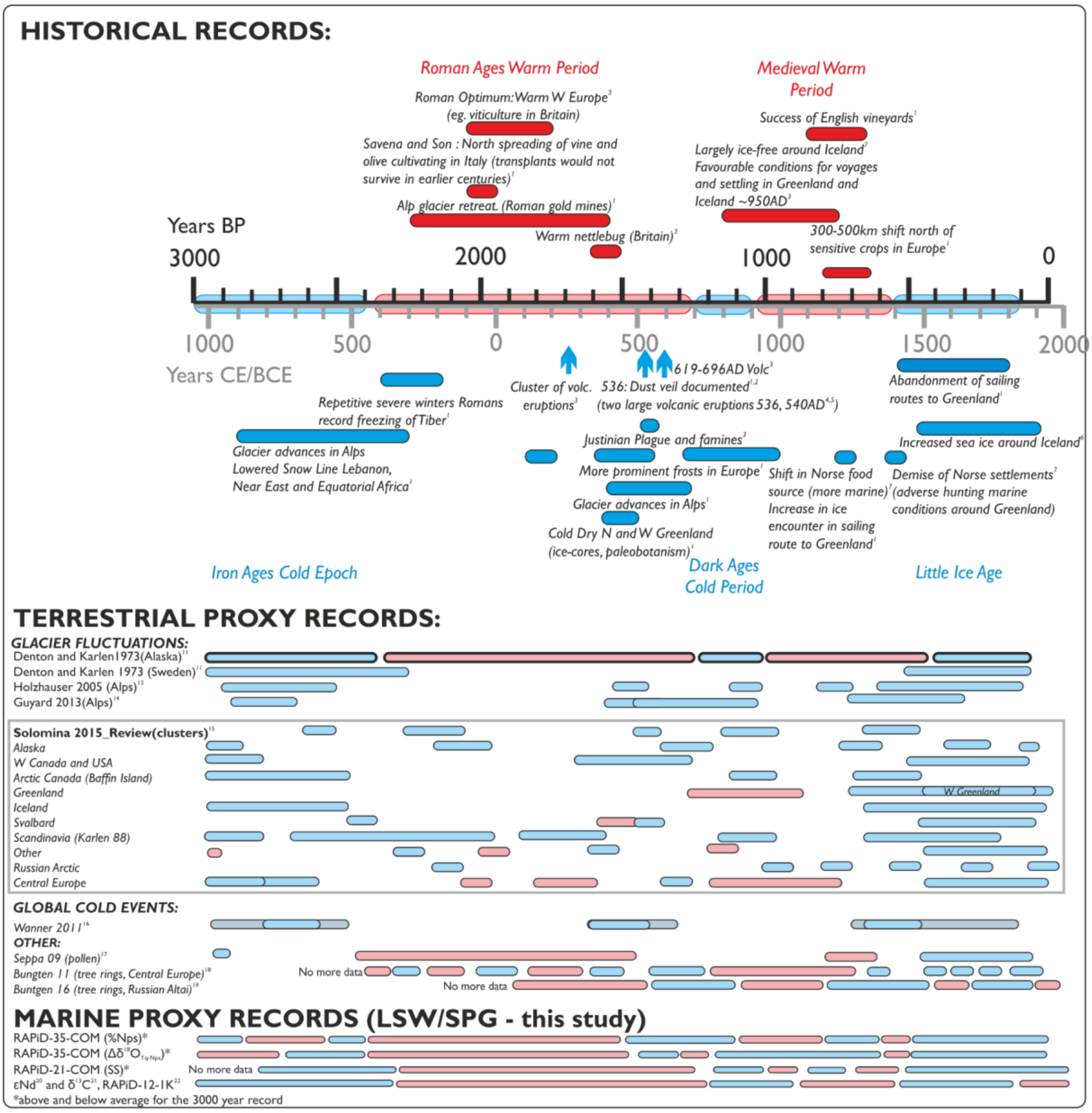

Supplementary Figure 4. Schematic comparison of the historical event timeline with the marine records (from this study) presented in Figure 4 (main text) with some terrestrial records. Red and blue lines denote the time-span for the evidence for warm and cold periods, respectively. Ages are in years BP (black) and years CE/BCE (grey). The historical information has been extracted from several publications indicated by the superscript in the annotations: 1. Ref.[3] and references herein; 2. Ref. [4]; 3. Ref. [5] and references herein; 4. Ref. [6]; 5. Ref. [7]; 6. Ref. [8]; 7. Ref. [9]. Terrestrial records include glacier fluctuations (blue denote advances and pink retreats as stated in the respective studies). Note that glacier dynamics are sensitive to climate changes with a predominant summer temperature control on mass balance and therefore glacier size in most high to mid latitude[10]. The first glacier record which is highlighted in bold[11] is the one used as a framework in this study following the Holocene climate review by ref.[12], the next two glacier reconstructions are from the Alps[13,14]. The glacier advance clusters were extracted from the review paper by ref. [15] and the circum-North Atlantic glacier advances and retreats were extracted from each geographical location (bound by a grey box). The results show how geographically varied the glacier dynamics are and highlighting the complexities in order to extract the common signals. However, from the compilation of the glacier fluctuation data two periods are found with a larger concentration of glacier advances around 3000-2500 years BP and from 700-50 years BP. There is an interval between these periods (2300-800years BP) which suggests

no consistent glacier advances and even if the records do not present retreats in the glaciers this is probably suggesting milder climatic conditions. Other global cold events are also presented in this Figure from a carefully selected number records including temperature, precipitation and glacier advance records presented in ref. [16]. We also include some terrestrial reconstructions spanning most of the last 3000 years indicative of temperatures in Europe based on pollen[17] and tree-ring temperature reconstructions[18,19]. This data compilation is by no mean exhaustive but aims to highlight the broad temporal agreement between the climate variability from terrestrial proxy and historical data and the Subpolar North Atlantic ocean variability from the new marine records presented in this study and previously published records[20-22]. As shown in Figure 4 of the main text, blue and pink indicate below and above average for the 3000 year records, and hence suggesting weaker/stronger SPG and LSW formation, respectively.

Supplementary References:

1 Jonkers, L., Brummer, G. J. A., Peeters, F. J. C., Van Aken, H. M. & De Jong, M. F. Seasonal stratification, shell flux, and oxygen isotope dynamics of leftcoiling N. pachyderma and T. quinqueloba in the western subpolar North Atlantic. *Paleoceanography* **25** (2010).

2 Yashayaev, I., Seidov, D. & Demirov, E. A new collective view of oceanography of the Arctic and North Atlantic basins. *Progress in Oceanography* **132**, 1-21, (2015).

3 Lamb, H. Climate: Present, Past and Future. vol. 2: Climatic History and the Future, 835 pp. *Methuen & Co, London,* (1977).

4 Stothers, R. B. & Rampino, M. R. Volcanic eruptions in the Mediterranean before AD 630 from written and archaeological sources. *Journal of Geophysical Research: Solid Earth* **88**, 6357-6371 (1983).

5 McCormick, M. *et al.* Climate change during and after the Roman Empire: reconstructing the past from scientific and historical evidence. *Journal of Interdisciplinary History* **43**, 169-220 (2012).

6 Larsen, L. B. *et al.* New ice core evidence for a volcanic cause of the AD 536 dust veil. *Geophysical Research Letters* **35** (2008).

7 Sigl, M. *et al.* Timing and climate forcing of volcanic eruptions for the past 2,500 years. *Nature* **523**, 543-549, (2015).

8 Koch, L. The east Greenland ice,. *Medd. Grønland, København* **130**, 1-374 (1945).

9 Arneborg, J., Lynnerup, N. & Heinemeier, J. Human diet and subsistence patterns in Norse Greenland AD c. 980—AD c. 1450: Archaeological interpretations. *Journal of the North Atlantic* **3**, 119-133, (2012).

10 Oerlemans, J. Extracting a Climate Signal from 169 Glacier Records. *Science* **308**, 675-677, doi:10.1126/science.1107046, (2005).

11 Denton, G. H. & Karlen, W. Holocene Climatic Variations--Their Pattern and Possible Cause. *Quaternary Research* **3**, 155-205, (1973).

12 Mayewski, P. A. *et al.* Holocene climate variability. *Quaternary Research* **62**, 243-255, (2004).

13 Guyard, H., Chapron, E., St-Onge, G. & Labrie, J. Late-Holocene NAO and oceanic forcing on high-altitude proglacial sedimentation (Lake Bramant, Western French Alps). *The Holocene* **23**, 1163-1172, (2013).

14 Holzhauser, H., Magny, M. & Zumbuühl, H. J. Glacier and lake-level variations in west-central Europe over the last 3500 years. *The Holocene* **15**, 789-801 (2005).

15 Solomina, O. N. *et al.* Holocene glacier fluctuations. *Quaternary Science Reviews* **111**, 9-34, (2015).

16 Wanner, H., Solomina, O., Grosjean, M., Ritz, S. P. & Jetel, M. Structure and origin of Holocene cold events. *Quaternary Science Reviews* **30**, 3109-3123, (2011).

17 Seppä, H., Bjune, A. E., Telford, R. J., Birks, H. & Veski, S. Last nine-thousand years of temperature variability in Northern Europe. *Climate of the Past* **5**, 523-535, (2009).

18 Büntgen, U. *et al.* Cooling and societal change during the Late Antique Little Ice Age from 536 to around 660 AD. *Nature Geoscience,* (2016).

19 Büntgen, U. *et al.* 2500 Years of European Climate Variability and Human Susceptibility. *Science* **331**, 578-582, (2011).

20 Copard, K. *et al.* Late Holocene intermediate water variability in the northeastern Atlantic as recorded by deep-sea corals. *Earth and Planetary Science Letters* **313**, 34-44 (2012).

21 Oppo, D. W., McManus, J. F. & Cullen, J. L. Palaeo-oceanography: Deepwater variability in the Holocene epoch. *Nature* **422**, 277-277, (2003).

22 Thornalley, D. J., Elderfield, H. & McCave, I. N. Holocene oscillations in temperature and salinity of the surface subpolar North Atlantic. *Nature* **457**, 711-714, (2009).
